# Comment on “Choroidal Thickness in Patients with Mild Cognitive Impairment and Alzheimer's Type Dementia”

**DOI:** 10.1155/2016/5392105

**Published:** 2016-09-06

**Authors:** Salih Uzun

**Affiliations:** Etimesgut Military Hospital, Department of Ophthalmology, 06790 Ankara, Turkey

We have read and reviewed the paper entitled “Choroidal Thickness in Patients with Mild Cognitive Impairment and Alzheimer's Type Dementia” by Bulut et al. with interest [1]. The authors demonstrated that choroidal thickness (CT) was significantly smaller both in patients with Alzheimer's type dementia and in patients with mild cognitive impairment when compared to healthy control subjects. They also found a significant correlation between mini-mental state examination score and CT.

However, CT shows a significant diurnal variation. The choroid could increase its thickness by 50% in an hour and quadruple its thickness in a few days [2]. Kee et al. showed that the choroid can thin very rapidly, by about 100 *μ*m in 3-4 h in young chicks [3]. Tan et al. measured CT in humans with two-hour intervals between 9:00 AM and 5:00 PM and determined significant differences in CT among all measurement points [4]. Tan et al. found mean diurnal amplitude of CT as 33.7 ± 21.5 *μ*m (range: 3 to 67 *μ*m). The change in CT was also correlated with changes in systolic blood pressure. Therefore, one might expect that the physiological fluctuation in CT measurements could affect the test results as well as the results of the statistical analysis. We recommend performing OCT measurements at the same time point of the day.

On the other hand, a number of local and systemic physiological/pathological conditions may affect CT [2]. Therefore, we ask the authors whether they took the systemic disease (such as hypertension and diabetes mellitus) status and use of drugs that could affect CT measurements into consideration. We also wonder about the sleep, smoking, and exercise statuses of the patients and consumption of alcohol or caffeinated/noncaffeinated beverages before OCT. We are also curious about whether the BMI of the patients was taken into consideration, as well as the results of their systemic blood pressure measurements and the lighting conditions of the test room, since all those parameters significantly affect CT [2].

Finally, we want to ask whether the authors found any correlation between CT and the duration of the disease in patients with Alzheimer's type dementia.
